# A decade of sustained selection pressure on two surface sites of the VP1 protein of Enterovirus A71 suggests that immune evasion may be an indirect driver for virulence

**DOI:** 10.1038/s41598-019-41662-8

**Published:** 2019-04-01

**Authors:** Ryan Roberts, Pinn Tsin Isabel Yee, Shama Mujawar, Chandrajit Lahiri, Chit Laa Poh, Derek Gatherer

**Affiliations:** 1 Division of Biomedical & Life Sciences, Faculty of Health & Medicine, Lancaster University, Lancaster, LA1 4YT UK; 2 Centre for Virus & Vaccine Research, School of Science & Technology, Sunway University, Bandar Sunway, Kuala Lumpur, Selangor 47500 Malaysia; 3 Department of Biological Sciences, Sunway University, Bandar Sunway, Kuala Lumpur, Selangor 47500 Malaysia

## Abstract

Enterovirus A71 (EV-A71) is an emerging pathogen in the *Enterovirus A* species group. EV-A71 causes hand, foot and mouth disease (HFMD), with virulent variants exhibiting polio-like acute flaccid paralysis and other central nervous system manifestations. We analysed all enterovirus A71 complete genomes with collection dates from 2008 to mid-2018. All sub-genotypes exhibit a strong molecular clock with omega (dN/dS) suggesting strong purifying selection. In sub-genotypes B5 and C4, positive selection can be detected at two surface sites on the VP1 protein, also detected in positive selection studies performed prior to 2008. Toggling of a limited repertoire of amino acids at these positively selected residues over the last decade suggests that EV-A71 may be undergoing a sustained frequency-dependent selection process for immune evasion, raising issues for vaccine development. These same sites have also been previously implicated in virus-host binding and strain-associated severity of HFMD, suggesting that immune evasion may be an indirect driver for virulence (154 words).

## Introduction

The genus *Enterovirus* (Order *Picornavirales*, Family *Picornaviridae*) contains many viruses pathogenic to humans. Historically, the most important have been the three human polioviruses, found within species *Enterovirus C*, but there are also several significant emerging pathogens^1^. Among these is Enterovirus A71 (EV-A71) which, along with around a dozen other viruses from species *Enterovirus A* and *Enterovirus B*^2^ causes hand, foot and mouth disease (HFMD). EV-A71 exhibits extensive genetic diversity, with genome sequences clustered into three established genotypes: A, B and C^3^. Genotypes B and C are also divisible into sub-genotypes: B1 to B5 and C1 to C5, respectively. A small number of outlier EV-A71 sequences have led to suggestions for the definition of additional genotypes and sub-genotypes^4,5^.

HFMD surveillance in China from 2008–2014 showed that EV-A71 was the major aetiological agent of the disease, in at least 44% of cases^6^. The milder end of the clinical spectrum of EV-A71 infection in children presents as high fever and vomiting, together with the characteristic HFMD pattern of ulceration on the palms of the hands, soles of the feet and within the mouth. At the severe end of the range, brainstem and cerebellar encephalitis complications arise^7^, along with an acute flaccid paralysis that has led EV-A71 to be dubbed “the new polio”^8^. Further complications of cardiopulmonary failure lead to death in the most severe cases. With several large HFMD outbreaks in east Asian countries over the last three decades, totalling several million cases and thousands of fatalities^9^, EV-A71 constitutes an emerging pathogen with epidemiological impact far beyond that of classic milder HFMD caused by other viruses. Although vaccine development for EV-A71 has been a research priority for nearly two decades^10,11^, there is no universally licensed vaccine or antiviral treatment for EV-A71 infection^12^.

A further research priority in recent years has been elucidation of the molecular basis of neurovirulence in EV-A71. Such knowledge would enable prediction of the likely severity of new outbreaks, or prognosis in individual cases where the viral genome sequence could be determined^13–19^. Searches for positive selection have also been carried out^20–23^ and there is some overlap between the detected residues in the two types of study, suggesting that the virulence of EV-A71 may be undergoing natural selection.

Evolution of EV-A71 at the molecular level has implications for vaccine design. EV-A71 sub-genotypes are serologically distinctive, implying that acquired immunity to one sub-genotype may not be completely protective in future outbreaks of different sub-genotypes^24^. Supportive of this notion, epidemics of EV-A71 often correlate with the appearance of a previously unencountered sub-genotype in a population - for instance the successive epidemics of sub-genotypes B3, B4 and B5 in Singapore^25^ and the cyclical nature of outbreaks of EV-A71^26^. Tee *et al*.^20^ show that EV-A71 phylogenetic trees display a ladder-like morphology, suggesting that immune evasion may be a selective pressure producing antigenic drift within EV-A71 cyclical sub-genotypes.

The preponderance of large epidemics of virulent EV-A71 in east Asia, despite the presence of the virus on all continents, is unexplained. Host factors such as HLA-A33 and HLA-DR17 responses have been linked to severe HFMD^27^, suggesting that human populations may differ in susceptibility. This may help to explain why separation of virulent and non-virulent strains purely on the basis of viral genome sequence, has remained elusive.

In this report, we update the analyses of Tee *et al*.^20^, which covered all EV-A71 sequences up to mid-2008, by analysing EV-A71 genome sequences from 2008 to mid-2018. We examine the behaviour of the molecular clock within sub-genotypes, and use Bayesian skyline analysis to demonstrate population dynamics of the two most important sub-genotypes of the last decade. We then search for evidence of amino acid residues under positive selection, and display their patterns of variation on phylogenetic trees. The structural consequences of positive selection on EV-A71 proteins are examined and implications for immune evasion, antigenic drift and virulence are discussed.

## Results

### Genetic distance between sub-genotypes

Genetic distance, measured by the Tamura-Nei substitution model^28^, averages at 0.30 to 0.33 substitutions per site, between EV-A71 genotypes. Within genotypes, the corresponding figure is 0.17 to 0.19 substitutions per site between sub-genotypes. The genetic distance from EV-A71 to CV-A16, EV-A71’s nearest relative within species *Enterovirus A*, ranges from 0.35 to 0.43 substitutions per site, and to baboon enterovirus A13, the outlier in *Enterovirus A*, from 0.85 to 0.91 substitutions per site (Table 1).

**Table 1:** Pairwise genetic distances between viruses of species *Enterovirus A* and subtypes of EV-A71.

	A	B0	B1	B2	B3	B4	B5	C1	C2	C3	C4	C5	A16
AB575911 EV-A71/B0	0.32												
AB575913 EV-A71/B1	0.31	0.17						B	0.17				
AB575927 EV-A71/B2	0.31	0.20	0.10					C	0.19				
DQ341367 EV-A71/B3	0.32	0.23	0.20	0.17							B	C	
DQ341366 EV-A71/B4	0.33	0.23	0.16	0.10	0.17					A	0.32	0.33	
KF974781 EV-A71/B5	0.32	0.23	0.17	0.12	0.19	0.09				B		0.30	
AB575935 EV-A71/C1	0.32	0.29	0.30	0.31	0.30	0.31	0.31						
AB575939 EV-A71/C2	0.33	0.29	0.31	0.31	0.31	0.32	0.31	0.10					
DQ341356 EV-A71/C3	0.33	0.30	0.30	0.32	0.32	0.31	0.32	0.12	0.11				
EU131776 EV-A71/C4	0.32	0.28	0.28	0.28	0.26	0.28	0.28	0.24	0.27	0.26			
EU527983 EV-A71/C5	0.35	0.30	0.31	0.31	0.33	0.31	0.32	0.16	0.19	0.20	0.27		
MG182694 CV-A16	0.35	0.39	0.39	0.40	0.42	0.40	0.42	0.41	0.43	0.43	0.42	0.42	
AF326750 Baboon A13	0.89	0.87	0.86	0.85	0.84	0.88	0.87	0.89	0.91	0.88	0.85	0.85	0.86

**Figure 1:**
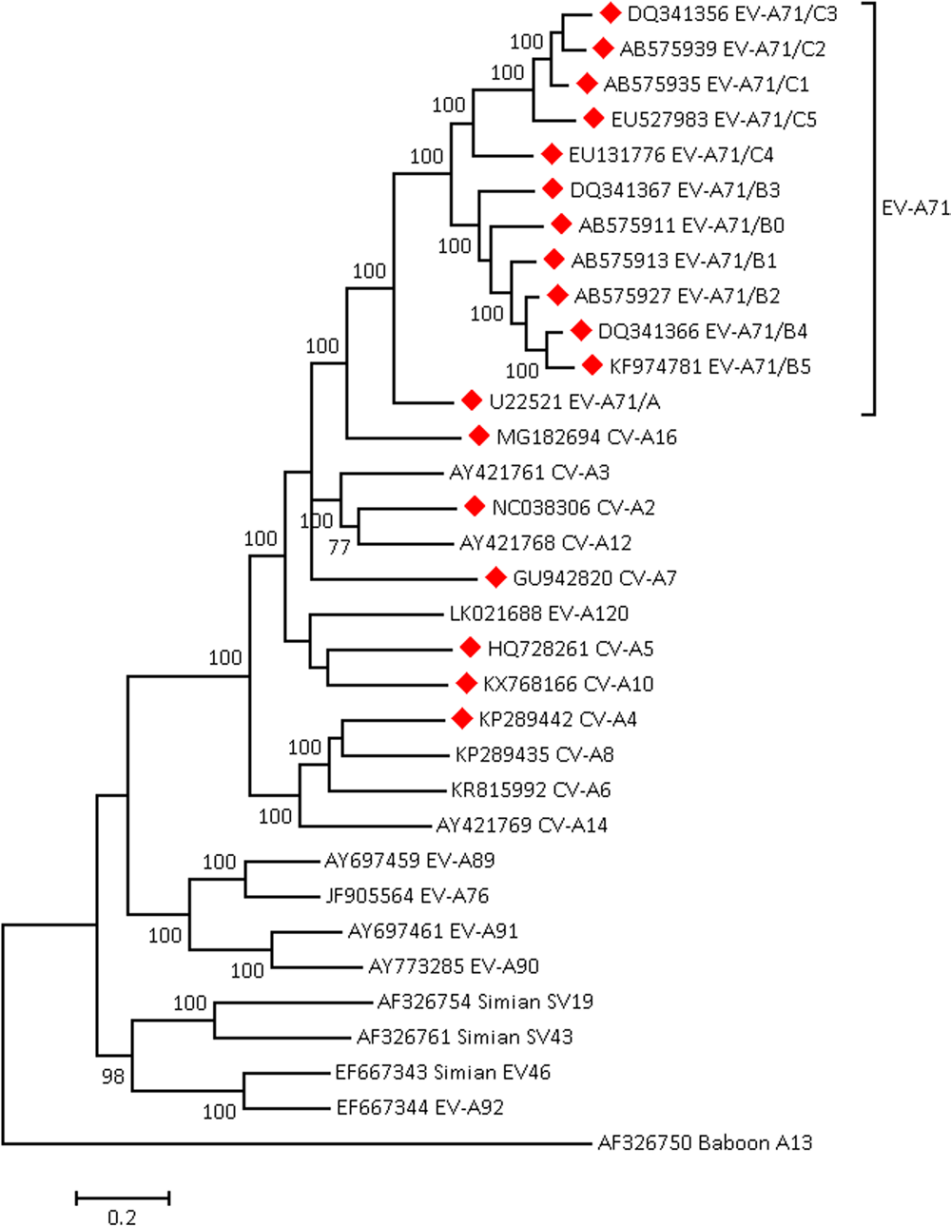
Phylogenetic relationships within species *Enterovirus A*. Maximum likelihood phylogenetic tree of reference genomes in the species *Enterovirus* A, and representative sub-genotype sequences of EV-A71, using the General Time Reversible model. A discrete Gamma distribution was used to model evolutionary rate differences among sites (5 categories, shape parameter = 0.5488). Bootstrap values are displayed at nodes. Red triangle: known virus causing HFMD. Scale: substitutions per site.

Genetic distances, in substitutions per site, calculated using the TN93 substitution matrix with sitewise rate variation modelled using a gamma distribution (shape parameter = 0.6868), between EV-A71 sub-genotypes, coxsackie virus CV-A16 (their nearest non-EV-A71 relative in *Enterovirus A*) and baboon A13, the outlier within *Enterovirus A* (see Fig. 1). The upper inset table shows the mean genetic distance within genotypes B and C, and the lower inset table shows the mean genetic distances between genotypes.

Figure 1 places Table 1 in the context of the wider *Enterovirus A* species, showing phylogenetic relationships within species *Enterovirus A* and indicating viruses known to cause HFMD. HFMD-associated viruses tend to be more closely related to EV-A71 but phylogenetic clustering is not complete, and members of species *Enterovirus B* (not included in Fig. 1) can also cause HFMD^2^.

### Molecular clocks in EV-A71 sub-genotypes

Table 2 summarizes qualifying EV-A71 sequences for each sub-genotype (see Methods section for qualification protocol). Since 2007, genotypes B5 and especially C4 have been predominant in GenBank.

**Table 2:** Qualifying sequences of EV-A71.

Genotype	Number of qualifying sequences	Date range	TempEst R	Number of post-2007 sequences	Slr omega (dN/dS)
A	1	2010	N/A	1	N/A
B0	2	1966	N/A	0	N/A
B1	12	1971–1986	0.996	0	N/A
B2	2	1986	N/A	0	N/A
B3	0	N/A	N/A	0	N/A
B4	10	2000–2011	N/A	6	N/D
B5	103	2003–2017	0.950	100	0.041
C1	8	1991–2010	N/D	3	N/A
C2	27	1998–2016	0.757	12	0.028
C3	0	N/A	N/A	N/A	N/A
C4	475	1998–2017	0.911	415	0.051
C5	10	2007–2012	0.937	9	N/D

Only sequences of >5 kb with a collection date were used. Sequences were removed if they were: a) annotated as unverified, b) annotated as cultured before sequencing, c) duplicates, d) found to have runs of unresolved bases, or e) adjudged to be recombinants (see Methods section 4.1). N/A: not applicable. N/D: not determined due to small sample size.

**Figure 2:**
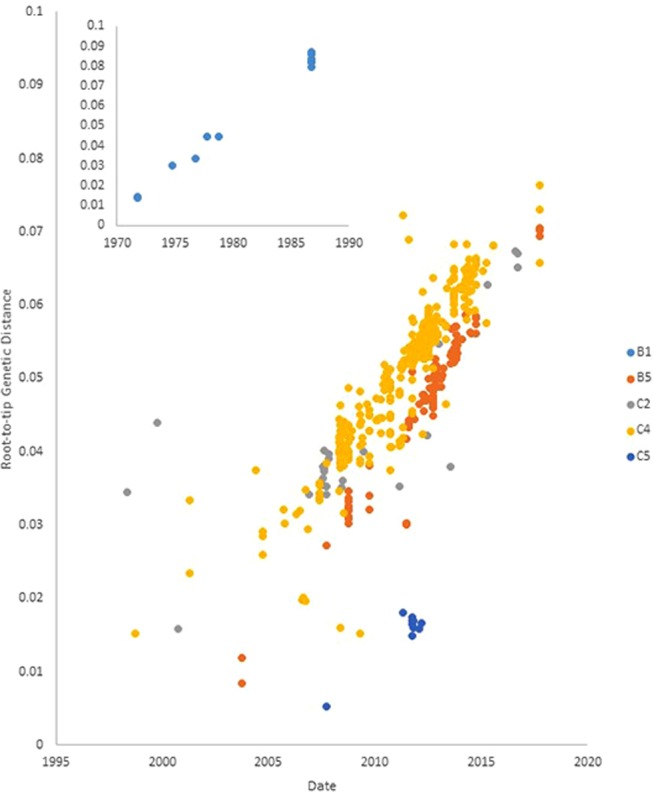
TempEst analysis of molecular clocks in genotypes B1, B5, C2, C4 and C5. x-axis: sampling dates; y-axis: root-to-tip distance on a maximum likelihood tree using the GTR + G substitution model. Sub-genotype B1 in inset. See Table 2 for correlation coefficient R values for each sub-genotype.

Figure 2 plots the correlation co-efficients for the root-to-tip distance on the input tree and sampling date. Sub-genotypes B1, B5, C4 and C5 have very high R values (>0.9, see Table 2) suggesting highly clock-like behaviour. The R value of C2 (0.76) is somewhat less than the others, but even this is strong enough to indicate clock-like behaviour. Bayesian analysis of clock models using BEAST shows that relaxed molecular clock models are the likeliest for all genotypes (see Supplementary Data) and these were used for the construction of Bayesian skylines on sub-genotypes B5 and C4.

### Bayesian skyline plots of EV-A71

**Figure 3:**
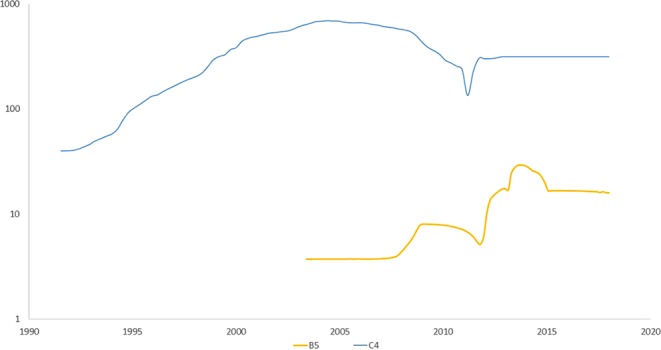
Bayesian skyline plots for sub-genotypes B5 and C4. Bayesian skylines were plotted in BEAST using the relaxed exponential clock model and the GTR + G substitution model; x-axis: collection date; y-axis: relative genetic diversity.

Figure 3 shows the Bayesian skyline plots for sub-genotypes B5 and C4. These sub-genotypes emerged in 2003 and 1998, respectively. BEAST analysis by Tee *et al*.^20^ indicated most recent common ancestor (MRCA) dates of 2001 for B5 and 1992 for C4. The Bayesian skylines suggest that B5 expanded around 2007 and then again from 2012 to 2014. C4, by contrast shows a steadier expansion from the early 1990s, peaking around 2004 with a decline in the early part of the present decade, coincident with the second expansion of B5.

### Natural selection on EV-A71

**Figure 4:**
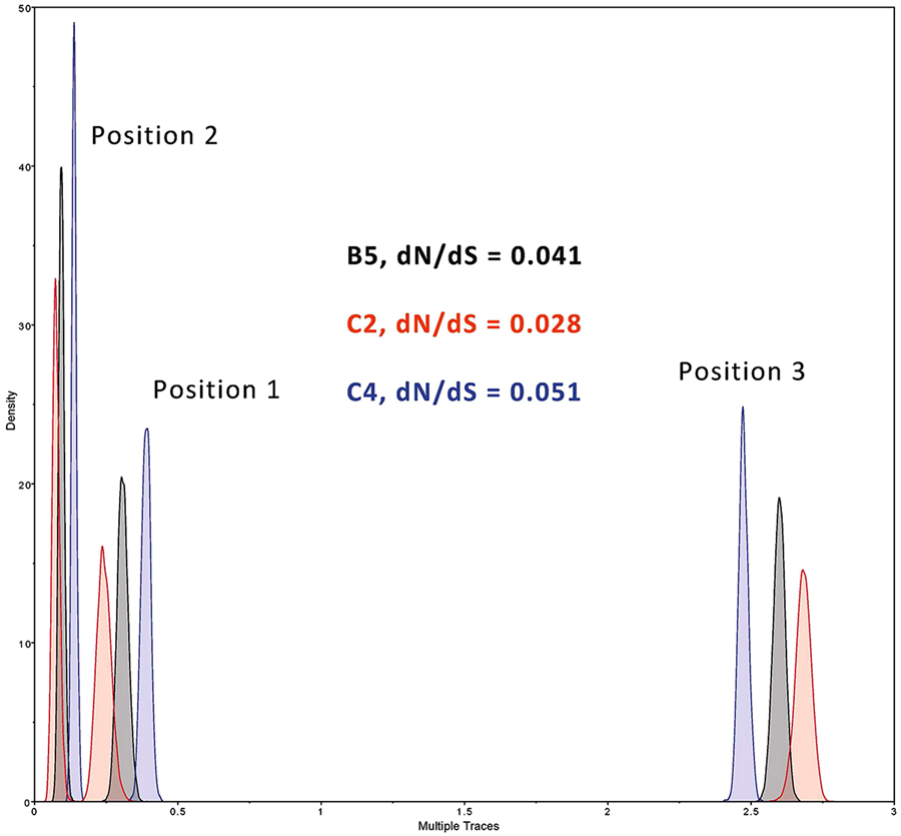
Substitution rates at the 3 codon positions, relative to the overall substitution rate, of sub-genotypes B5, C2 and C4 since 2008. Substitution rates for all three reading frames were calculated in BEAST using the relaxed exponential clock model and the GTR + G substitution model, then plotted in Tracer. Grey curves: B5; red curves: C2; blue curves: C4.

Table 2 and Fig. 4 show the omega (dN/dS) values calculated for sub-genotypes B5, C2 and C4 collected since 2008. Figure 4 also shows the relative substitution rates in the 3 codon positions. Together, these indicate that EV-A71 has been evolving for the past decade mostly under selective constraint.

**Figure 5:**
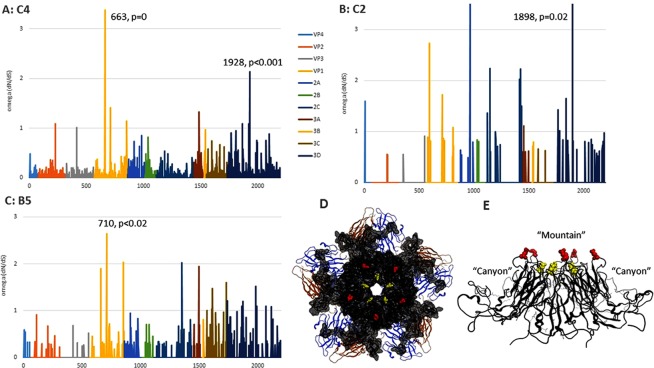
Positive selection in sub-genotypes B5, C2 and C4 since 2008. For panels (A) (sub-genotype C4), (**B**) (sub-genotype C2) and (**C**) (sub-genotype B5), x-axis: amino acid position in polyprotein (colours indicate consecutively the genes for VP4, VP2, VP3, VP1, 2 A, 2B, 2 C, 3 A, 3B, 3 C, 3D); y-axis: omega (dN/dS) calculated for each position in Slr. Positions crossing the statistical significance threshold are indicated and their level of statistical significance shown. Panel D shows the solved structure of the VP1/VP2/VP3 complex of a sub-genotype C4 strain (PDB accession 4YVS); red residues: position 663; yellow residues: position 710. The backbone and surface of the VP1 protein are in black, VP2 backbone in brown and VP3 in blue. Panel E shows the VP1 protein tilted 90 degrees to illustrate the location of residues 663 and 710 in the “mountain” region.

Within this general pattern of selective constraint, positive selection is detected on certain sites. Figure 5 shows the sitewise omega Slr output for sub-genotypes B5, C2 and C4. Eight sites in sub-genotype C4, fifteen sites in C2 and fourteen sites in B5 have omega values >1, suggesting positive selection. However, only two positions in C4 are statistically significant, and one each in C2 and B5.

### Structural correlates of positive selection

Figure 5D shows the location of the selected site in the VP1 protein of PDB structure 4YVS (sub-genotype C4). VP1, VP2 and VP3 make up the icosahedral surface structure of the EV-A71 virus particle. Five VP1 proteins form a pentamer which has a raised “mountain” feature^29,30^ at its vertex. Between the “mountain” and another raised feature is a “canyon”. An antibody binding site is found on both slopes of the canyon^31^.

### Temporal pattern of positive selection

**Figure 6:**
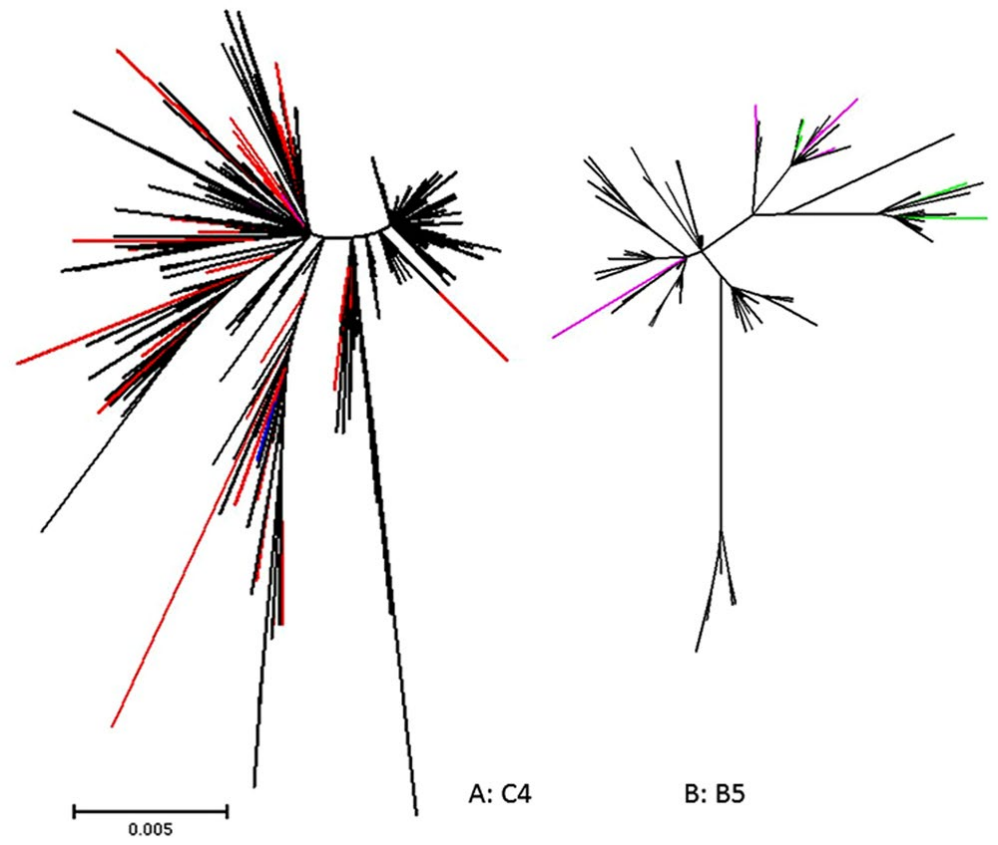
Maximum likelihood unrooted trees using a Poisson distance amino acid alignment showing toggling of the positively selected sites in VP1 during the evolution of sub-genotypes C4 and B5 since 2008. (**A**) C4 site 663 (VP1–98), black branches are E, red K, mauve G, blue V. (**B**) B5 site 710 (VP1–145), black branches are E, mauve G, green Q. Scale is amino acid substitutions per site. Site 663 in sub-genotype C4 toggles between amino acids E, K, G and V. Site 710 in sub-genotype B5 toggles between amino acids E, G and Q.

Figure 6 shows the toggling of amino acids at statistically significant positively selected sites 663 in sub-genotype C4 and 710 in B5 since 2008.

## Discussion

Our sampling protocol for EV-A71 sequences used in this analysis, is directed at all complete genomes over the last decade, where collection dates are available. This reduces the 12122 total sequences from EV-A71 in GenBank on our sequence retrieval date to a subset of 697, from which a further 46 suspected recombinant genomes were removed. The stringency of this protocol is designed to ensure data quality, but carries the risk of sampling bias. We note that Table 1 presents overwhelmingly B5 and C4 sub-genotypes. However, we suggest that this is not a result of any lack of interest by sequencing laboratories in other sub-genotypes but is rather more likely to be an indication of genuine predominance of these sub-genotypes over the study decade. Nevertheless, we acknowledge, in the absence of any globally co-ordinated monitoring programme for EV-A71, that the potential for inadequate sampling remains a shortcoming of this study.

Although EV-A71 and influenza A are very different viruses, being positive-strand unsegmented and negative-strand segmented, respectively, several factors may be identified that suggest that their epidemiological dynamics may be similar. For instance, we show in this paper that EV-A71 sub-genotypes exhibit remarkably regular clock-like substitution behaviour (Fig. 2), comparable to that shown by seasonal influenza A subtypes^32^, as previously suggested by Tee *et al*.^20^. We calculate that overall rates of omega (dN/dS) in EV-A71 sub-genotypes have been low since 2008 (Table 2, Fig. 4), but we also find evidence of a small number of sites under positive selection, with omega >1 (Fig. 5). Low overall omega implies strong purifying selection and a virus that is well adapted to its human hosts. This has previously been found in a study restricted to the evolution of the VP1 gene^23^. Figure 5 is notable for many sites presenting signals of positive selection that do not achieve statistical significance. This may be due to more fleeting selection pressures that are not sustained over the entire decade examined in the analysis.

Again as in influenza A, we find that those sites with statistically significant positive selection tend to be in prominent functional surface positions^31,33^. Together with the ladder-like phylogenetic trees of EV-A71^20^, these factors suggest an evolutionary scenario of a proficiently host-adapted virus engaged in intense immune evasion mediated at a small number of virus particle surface sites that interact directly with the host immune system. The result of this evolutionary struggle between virus and host is antigenic drift, with annual seasonal epidemics of influenza A, and cycling non-seasonal EV-A71 outbreaks^20,26^. When novel genotypes are formed – either by reassortment in influenza or recombination and cladogenesis in EV-A71 – there may be a partial absence of cross-protectivity derived from previous genotypes, allowing surges in new sub-genotype cases. For EV-A71, this is illustrated using Bayesian skyline analysis for pre-2008 genotypes by Tee, *et al*.^20^ and for post-2008 sub-genotypes by Fig. 3. Our skyline analysis shows a decline of C4 in the early part of the present decade after its peak around 2004, and that this may be coincident with the second expansion of B5, suggesting competition among sub-genotypes for hosts.

The pattern of natural selection in EV-A71 has the added complication that the positively selected sites may also have been involved in virulence as well as antibody binding. Polyprotein positions 663, 710 and 729 have previously been implicated in changes in cell tropism^34^, and also in virulence^15^. The first two of these sites are among the identified positively selected sites in the present study and are also among the positively selected sites previously detected^20,21^. Specifically, amino acids G, Q, E and R at position 710 have been associated with virulence^13,16,17,19^ with Q and R correlated to acute flaccid paralysis^18^. When Q is present at position 710, experimental evidence suggests it may confer better virus-cell binding and also immune evasion^18^, where E is present it may confer higher viraemia and neurovirulence^19^. At position 663, K is associated with the development of acute flaccid paralysis^18^.

We find that in sub-genotype C4 genomes since 2008, K is the second most common residue, after E, at positively selected site 663 (Fig. 6A, red branches for K), implying the frequent circulation of flaccid paralysis-inducing strains. We also find that in B5 genomes since 2008, residue Q is occasionally present at positively selected site 710 (Fig. 6B, green branches for Q), implying that this sub-genotype may also pose an ongoing flaccid paralysis risk. Moreover, all the residues we see at position 710 in genotype B5 since 2008 have been implicated in some aspect of increased virulence. Figure 6 also shows that variation at positions 663 and 710 since 2008 exhibits a toggling pattern, with many tip substitutions on the phylogenetic trees. This indicates that the signal for positive selection is not due to a past selective sweep of EV-A71, but rather suggests a sustained frequency-dependent selection process, where antigenic novelty is favoured, but only as long as it takes for the host population to develop herd immunity against the new variant. The continuity of position of positively selected sites in both post-2008 genomes and in previous studies^20,21^ shows that the nature of the molecular interaction between host and virus producing the selective pressure has been unchanged over a timescale of decades. Both position 663 and 710 have the same predominant variant, E. Indeed, the basal position of E in both trees in Fig. 6 may indicate that E is the most functionally efficient residue at these positions from the point of view of the action of VP1 as coat protein, and that it may only be its vulnerability to antibody binding that selects for periodic temporary substitution to a small handful of variants. The involvement of these same residues in virulence may be due to a dependency of virulence on the efficiency of virus binding to host cells^34^. This implies that virus immune evasion may be driving changes in virulence, by an incidental effect on basic virus-host interaction.

One final comparison with seasonal influenza is that any future approved EV-A71 vaccines may need to be renewed on a regular basis, as positive selection will produce vaccine resistant forms. However, unlike seasonal influenza, where the repertoire of antigenic variation is large, the relatively small set of immune evasion options at residues 663 and 710 displayed by EV-A71 over the last decade and in previous studies, suggests that a cocktail vaccine composed of strains with the seven variant residues may generate a herd immunity that will last several years.

## Methods

### Collection and processing of Enterovirus A71 genome sequences

On 3rd October 2018, GenBank contained 12122 nucleotide sequences classified as from Enterovirus A71. Of these, 877 were greater than 5 kb in length. These were downloaded and sequences annotated as passaged in cell culture were manually removed, along with those annotated as unverified, or with internal runs of more than 6 consecutive Ns, reducing the number of qualifying sequences to 784.

Sequences with a collection date were then extracted using *tempus et locus* (TeL^35^). 697 qualifying sequences were found to have collection dates. Rapid input of sequences sets to TempEst for molecular clock analysis is assisted by the following expedient. Where only year of collection is given, TeL generated a collection date of 30^th^ June for that year (155 cases; no sequences had genuine collection dates on the 30^th^ June of any year). Where only a month and year of collection is given, TeL generated the 15^th^ of that month (111 cases; 12 sequences with genuine collection dates on the 15^th^ of any month were noted).

TeL-dated output sequences were grouped into sub-genotypes using *E-type* (available in the Supplementary Data), which compares each sequence with a subset of the reference genomes given by van der Sanden, *et al*.^36^ plus MG672478^37^ for putative genotype E, using *needle* from EMBOSS^38^ to perform a global pairwise alignment. The *needle* output is parsed for each reference genome and the closest match was used to assign a sub-genotype to the query sequence. As output, *E-type* produced a separate FASTA-formatted file for each sub-genotype. Where the difference between the closest sub-genotype match and the second closest match for any sequence was < = 5% (152 sequences), further analysis was carried out using Simplot^39^. If Simplot indicated a likely recombination event, the sequence was removed from the analysis (46 sequences).

Duplicate sequences are also removed from each sub-genotype file if they share the same collection date, using *duptrimmer* (available in the Supplementary Data). This is a rare occurrence, found once in genotype B5 and twice in C4.

### Phylodynamic analysis of sub-genotype sets

Sub-genotypes output files from *E-type* with ten or more qualifying sequences after the removal of duplicates and recombinants, were aligned using MAFFT^40^, and a maximum likelihood tree produced in MEGA^41^ using the GTR + G substitution model^42^. TempEst^32^ was used to derive the best fitting root and assess clock-like behaviour for each sub-genotype tree. For those sub-genotypes with more than 25 qualifying sequences (only C4 and B5), Bayesian skyline plots were produced using BEAST^43^ and Tracer^44^.

### Detection of positive selection using Slr

Genome sequences with a collection date before 2008 were removed and each sub-genotype alignment was trimmed to the boundaries of the coding sequence. Where necessary, alignment was refined using Muscle in “align codons” mode within MEGA. Positive selection, neutrality and constraint were analysed on a site-wise basis using Slr^45^ (version 1.5.0). An initial estimate of the transition/transversion bias (kappa) was obtained in MEGA and was used along with an initial value of omega (dN/dS) of 1 (corresponding to neutral evolution) in the Slr input parameters. After the first run, the values of kappa and omega derived by Slr were used as input parameters to confirm convergence. Visualization of positively detected sites was performed in Molecular Operating Environment (MOE), 2013.08 (Chemical Computing Group ULC, 1010 Sherbooke St. West, Suite #910, Montreal, QC, Canada, H3A 2R7).

## References

[1] Zell R (2017). ICTV Virus Taxonomy Profile: Picornaviridae. J Gen Virol.

[2] Li Y, Zhu R, Qian Y, Deng J (2012). The characteristics of blood glucose and WBC counts in peripheral blood of cases of hand foot and mouth disease in China: a systematic review. PLoS One.

[3] Brown BA, Oberste MS, Alexander JP, Kennett ML, Pallansch MA (1999). Molecular epidemiology and evolution of enterovirus 71 strains isolated from 1970 to 1998. J Virol.

[4] Castro CM, Cruz AC, Silva EE, Gomes Mde L (2005). Molecular and seroepidemiologic studies of Enterovirus 71 infection in the State of Para, Brazil. Rev Inst Med Trop Sao Paulo.

[5] Yip CC (2013). Genetic characterization of EV71 isolates from 2004 to 2010 reveals predominance and persistent circulation of the newly proposed genotype D and recent emergence of a distinct lineage of subgenotype C2 in Hong Kong. Virol J.

[6] Liu W (2015). Spatiotemporal Dynamics of Hand-Foot-Mouth Disease and Its Relationship with Meteorological Factors in Jiangsu Province, China. PLoS One.

[7] Wang MG, Sun HM, Liu XM, Deng XQ (2017). Clinical analysis of 59 children with hand foot and mouth diseases due to enterovirus EV71 and concomitant viral encephalitis. Eur Rev Med Pharmacol Sci.

[8] Teoh HL, Sampaio H, Farrar M (2015). Enterovirus 71 Neuroimaging: “The New Polio of the 21st Century”. Pediat Therapeut.

[9] Xing W (2014). Hand, foot, and mouth disease in China, 2008-12: an epidemiological study. Lancet Infect Dis.

[10] Lin YC, Wu CN, Shih SR, Ho MS (2002). Characterization of a Vero cell-adapted virulent strain of enterovirus 71 suitable for use as a vaccine candidate. Vaccine.

[11] Wang, X. *et al*. Structure, Immunogenicity, and Protective Mechanism of an Engineered Enterovirus 71-Like Particle Vaccine Mimicking 80S Empty Capsid. *J Virol* **92** (2018). 10.1128/JVI.01330-17 PMC5730765 29070691

[12] Yee PTI, Poh CL (2017). Impact of genetic changes, pathogenicity and antigenicity on Enterovirus- A71 vaccine development. Virology.

[13] Li R, Zou Q, Chen L, Zhang H, Wang Y (2011). Molecular analysis of virulent determinants of enterovirus 71. PLoS One.

[14] Chang SC (2012). Genetic characterization of enterovirus 71 isolated from patients with severe disease by comparative analysis of complete genomes. J Med Virol.

[15] Liu Y (2014). A novel finding for enterovirus virulence from the capsid protein VP1 of EV71 circulating in mainland China. Virus Genes.

[16] Caine EA, Moncla LH, Ronderos MD, Friedrich TC, Osorio JE (2016). A Single Mutation in the VP1 of Enterovirus 71 Is Responsible for Increased Virulence and Neurotropism in Adult Interferon-Deficient Mice. J Virol.

[17] Huang SW (2015). Mapping Enterovirus A71 Antigenic Determinants from Viral Evolution. J Virol.

[18] Huang YP, Lin TL, Lin TH, Wu HS (2013). Antigenic and genetic diversity of human enterovirus 71 from 2009 to 2012, Taiwan. PLoS One.

[19] Kataoka C (2015). The Role of VP1 Amino Acid Residue 145 of Enterovirus 71 in Viral Fitness and Pathogenesis in a Cynomolgus Monkey Model. PLoS Pathog.

[20] Tee KK (2010). Evolutionary genetics of human enterovirus 71: origin, population dynamics, natural selection, and seasonal periodicity of the VP1 gene. J Virol.

[21] Chen X (2010). Analysis of recombination and natural selection in human enterovirus 71. Virology.

[22] Wang HY (2010). Inferring nonneutral evolution from contrasting patterns of polymorphisms and divergences in different protein coding regions of enterovirus 71 circulating in Taiwan during 1998–2003. BMC Evol Biol.

[23] Chan YF (2012). Comparative genetic analysis of VP4, VP1 and 3D gene regions of enterovirus 71 and coxsackievirus A16 circulating in Malaysia between 1997–2008. Trop Biomed.

[24] van der Sanden SM (2016). Prediction of Protection against Asian Enterovirus 71 Outbreak Strains by Cross-neutralizing Capacity of Serum from Dutch Donors, The Netherlands. Emerg Infect Dis.

[25] Chan KP (2003). Epidemic hand, foot and mouth disease caused by human enterovirus 71, Singapore. Emerg Infect Dis.

[26] McMinn PC (2002). An overview of the evolution of enterovirus 71 and its clinical and public health significance. FEMS Microbiol Rev.

[27] Chang LY (2008). HLA-A33 is associated with susceptibility to enterovirus 71 infection. Pediatrics.

[28] Tamura K, Nei M (1993). Estimation of the number of nucleotide substitutions in the control region of mitochondrial DNA in humans and chimpanzees. Mol Biol Evol.

[29] Smith TJ, Olson NH, Cheng RH, Chase ES, Baker TS (1993). Structure of a human rhinovirus-bivalently bound antibody complex: implications for viral neutralization and antibody flexibility. Proc Natl Acad Sci USA.

[30] Smith TJ (1993). Structure of human rhinovirus complexed with Fab fragments from a neutralizing antibody. J Virol.

[31] Lee H (2013). A strain-specific epitope of enterovirus 71 identified by cryo-electron microscopy of the complex with fab from neutralizing antibody. J Virol.

[32] Rambaut A, Lam TT, Max Carvalho L, Pybus OG (2016). Exploring the temporal structure of heterochronous sequences using TempEst (formerly Path-O-Gen). Virus Evol.

[33] Koel BF (2013). Substitutions near the receptor binding site determine major antigenic change during influenza virus evolution. Science.

[34] Nishimura Y (2013). Enterovirus 71 binding to PSGL-1 on leukocytes: VP1-145 acts as a molecular switch to control receptor interaction. PLoS Pathog.

[35] Carter, A. R. & Gatherer, D. Tempus et Locus: a tool for extracting precisely dated viral sequences from GenBank, and its application to the phylogenetics of primate erythroparvovirus 1 (B19V). *bioRxiv*, 10.1101/061697 (2016).

[36] van der Sanden S, Koopmans M, Uslu G, van der Avoort H (2009). & Dutch Working Group for Clinical, V. Epidemiology of enterovirus 71 in the Netherlands, 1963 to 2008. J Clin Microbiol.

[37] Fernandez-Garcia MD (2018). Genetic Characterization of Enterovirus A71 Circulating in Africa. Emerg Infect Dis.

[38] Rice P, Longden I, Bleasby A (2000). EMBOSS: the European Molecular Biology Open Software Suite. Trends Genet.

[39] Lole KS (1999). Full-length human immunodeficiency virus type 1 genomes from subtype C-infected seroconverters in India, with evidence of intersubtype recombination. J Virol.

[40] Katoh, K., Rozewicki, J. & Yamada, K. D. MAFFT online service: multiple sequence alignment, interactive sequence choice and visualization. *Brief Bioinform* (2017). 10.1093/bib/bbx108 PMC6781576 28968734

[41] Kumar S, Nei M, Dudley J, Tamura K (2008). MEGA: a biologist-centric software for evolutionary analysis of DNA and protein sequences. Brief Bioinform.

[42] Tavaré S (1986). Some Probabilistic and Statistical Problems in the Analysis of DNA Sequences. Lectures on Mathematics in the Life Sciences.

[43] Drummond AJ, Rambaut A (2007). BEAST: Bayesian evolutionary analysis by sampling trees. BMC Evol Biol.

[44] Rambaut A, Drummond AJ, Xie D, Baele G, Suchard MA (2018). Posterior Summarization in Bayesian Phylogenetics Using Tracer 1.7. Syst Biol.

[45] Massingham T, Goldman N (2005). Detecting amino acid sites under positive selection and purifying selection. Genetics.

